# RETRACTION: Effect of Endoscopic Mucosal Resection and Endoscopic Submucosal Dissection on Postoperative Wound Bleeding‐Related Complications in Patients With Superficial Esophageal Cancer: A Meta‐Analysis

**DOI:** 10.1111/iwj.70399

**Published:** 2025-03-21

## Abstract

**RETRACTION:** LiuD. , LuoK. , ZhouX. , and YangS. , “Effect of Endoscopic Mucosal Resection and Endoscopic Submucosal Dissection on Postoperative Wound Bleeding‐Related Complications in Patients With Superficial Esophageal Cancer: A Meta‐Analysis,” International Wound Journal 21, no. 2 (2024): e14702, 10.1111/iwj.14702.PMC1096186338093697

The above article, published online on 30 January 2024, in Wiley Online Library (http://onlinelibrary.wiley.com/), has been retracted by agreement between the journal Editor in Chief, Professor Keith Harding; and John Wiley & Sons Ltd. Following an investigation by the publisher, all parties have concluded that this article was accepted solely on the basis of a compromised peer review process. The editors have therefore decided to retract the article. The authors did not respond to our notice regarding the retraction.

**RETRACTION:** D. Liu , K. Luo , X. Zhou , and S. Yang , “Effect of Endoscopic Mucosal Resection and Endoscopic Submucosal Dissection on Postoperative Wound Bleeding‐Related Complications in Patients With Superficial Esophageal Cancer: A Meta‐Analysis,” International Wound Journal 21, no. 2 (2024): e14702, 10.1111/iwj.14702.PMC1096186338093697

The above article, published online on 30 January 2024, in Wiley Online Library (http://onlinelibrary.wiley.com/), has been retracted by agreement between the journal Editor in Chief, Professor Keith Harding; and John Wiley & Sons Ltd. Following an investigation by the publisher, all parties have concluded that this article was accepted solely on the basis of a compromised peer review process. The editors have therefore decided to retract the article. The authors did not respond to our notice regarding the retraction.

